# Ionic Polymethacrylate Based Delivery Systems: Effect of Carrier Topology and Drug Loading

**DOI:** 10.3390/pharmaceutics11070337

**Published:** 2019-07-15

**Authors:** Dorota Neugebauer, Anna Mielańczyk, Rafał Bielas, Justyna Odrobińska, Maria Kupczak, Katarzyna Niesyto

**Affiliations:** Faculty of Chemistry, Department of Physical Chemistry and Technology of Polymers, Silesian University of Technology, 44-100 Gliwice, Poland

**Keywords:** polymer carriers, drug delivery, conjugates, self-assemblies, star polymers, graft polymers, poly(ionic liquid)s

## Abstract

The presented drug delivery polymeric systems (DDS), i.e., conjugates and self-assemblies, based on grafted and star-shaped polymethacrylates have been studied for the last few years in our group. This minireview is focused on the relationship of polymer structure to drug conjugation/entrapment efficiency and release capability. Both graft and linear polymers containing trimethylammonium groups showed the ability to release the pharmaceutical anions by ionic exchange, but in aqueous solution they were also self-assembled into nanoparticles with encapsulated nonionic drugs. Star-shaped polymers functionalized with ionizable amine/carboxylic groups were investigated for drug conjugation via ketimine/amide linkers. However, only the conjugates of polybases were water-soluble, giving opportunity for release studies, whereas the self-assembling polyacidic stars were encapsulated with the model drugs. Depending on the type of drug loading in the polymer matrix, their release rates were ordered as follows: Physical ≥ ionic > covalent. The studies indicated that the well-defined ionic polymethacrylates, including poly(ionic liquid)s, are advantageous for designing macromolecular carriers due to the variety of structural parameters, which are efficient for tuning of drug loading and release behavior in respect to the specific drug interactions.

## 1. Introduction

Conventional drug delivery formulations have significantly contributed to the effectiveness of disease treatment. Nevertheless, there is still a strong need to create modern carriers, including polymers, which are supposed to improve control of pharmacokinetics and biodistribution of “classic” and new drugs, their selective accumulation with reduced side effects, and enhanced effectiveness of therapeutic treatment. The progress in drug delivery has been advanced by the use of polymeric carriers for noninvasive and spatiotemporal release of different therapeutics [1,2]. The functional materials [3] with great biocompatibility, and optional biodegradability [4], are based on the “tailor-made” polymers with the well-defined hydrophilic/hydrophobic balance, particle size, or electric charge distribution. In contrast to linear polymers, the branched topology offers a broader spectrum of structural parameters, such as length and number of grafts/arms, and higher content of reactive groups, which can be used to adjust physicochemical properties responsible for efficiency of drug introduction and delivery. The polymers with sophisticated architectures, like star-shaped [5] and graft copolymers [6,7], are provided, by strategy of macromolecular engineering [8], to design carriers for drug delivery systems (DDS) with programmed activities, including controlled drug release profile, specific targeting to diseased tissues, and prolonged release time.

The nanosized DDS are classified into drug conjugates [9,10] and self-assembling systems [11]. The conjugate formation requires suitable functionalities in the polymer to attach bioactive compounds by covalent bonding, whereas proper hydrophilic–hydrophobic balance in the amphiphilic polymer affords the self-assembling behavior in aqueous solution resulting in micellar and aggregate superstructures with capability of drug entrapment via physical interactions [12]. The amphiphilic polymer conjugates can also be designed to provide dual DDS [13,14], containing two drugs loaded with various strength into the polymer matrix (conjugation vs. encapsulation) in additional respect to both drug and polymer nature. The interesting alternative is drug attachment via ionic bonding, which is weaker than the covalent bond due to electrostatic interactions between ions with opposite charges, but it seems to be more stable than the physical interactions. This variant requires the use of ionic polymers, including poly(ionic liquid)s (PIL) [15], where the counterions can be biologically active.

Both star-shaped [5,16] and graft copolymers [6,17] are convenient for introduction of multiple terminal active/functional groups, which can be used as conjugation sites. Generally, the nonlinear polymers form more stable micelles, which are characterized by longer time release of the drug than that of linear block copolymers [18]. Due to this, the latter have been stabilized by cross-linking in the core and/or shell (e.g., the doughnut shape micelles) [19]. The stability of micelles based on graft or star copolymers has been developed by hydrophobic–hydrophilic block structures of side chains/arms, which yielded amphiphilic core–shell cylindrical brushes [20], miktobrushes [21], or scorpion-like polymers [22]. These macromolecules, with the well-organized hydrophobic inner surrounded by hydrophilic outer layer, exhibited low critical micelle concentrations (CMC) and higher drug loading capacity compared with micelles of linear block copolymers [23]. It has also been reported that the star-shaped copolymers, in comparison to their linear analogs with similar molecular weight and composition, exhibit lower solution viscosity and smaller hydrodynamic radius, which is beneficial in excretion of system after drug release [24]. Additionally, in the case of the presence of polyester segments, which were shorter due to branching into the arms, the lower crystallinity improved control of degradation in correlation with the enhanced drug release [25]. Another advantage of polymers containing acidic units [26] has been indicated by pH activated drug release, i.e., significantly faster release at pH below 7.4 than at neutral pH (37 °C), which was observed for micellar systems of block copolymers grafted with 2-alkanone chains via acid-sensitive linker providing pH-dependent degradation [27]. The pH-dependent systems were also investigated for graft copolymers containing acidic units in the backbone or in the side chains [20], as well as for star copolymers with polyacidic segments in the arms [28]. Moreover, disability of self-assembling for some amphiphilic linear copolymers has been efficiently solved by their grafting onto polymer backbone [29].

**Figure 1:**
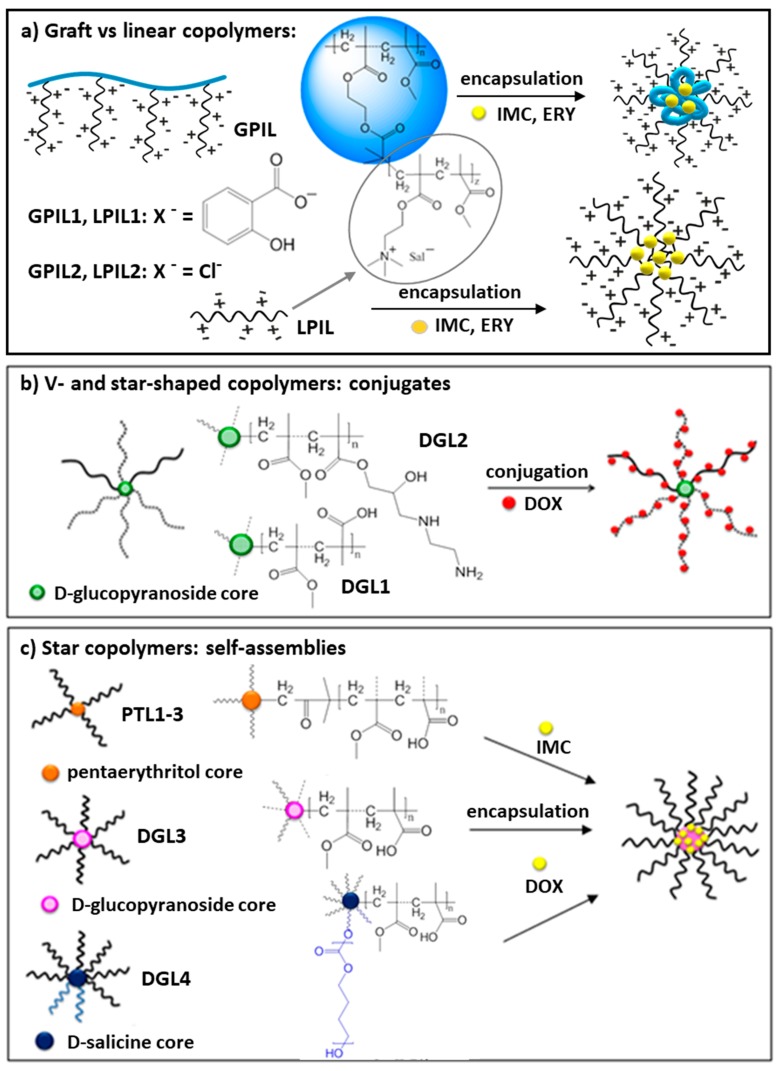
Drugs carried by grafted poly(ionic liquid)s (GPIL) (**a**), and star shaped polymers (DGL, PTL) (**b**,**c**), in the form of conjugates and self-assemblies.

In recent years our work was focused on the non-linear polymethacrylates designed for the nanosized DDS, including conjugates and self-assemblies (Figure 1). These studies provided better understanding of the influence of the hydrophobic/hydrophilic content on physicochemical and delivery properties of polymer carriers, which were varied by topology (graft vs. linear and stars vs. miktostars) and architecture (grafting degree, length of backbone and side chains or number and length of arms, core type) to regulate physical entrapment or chemical attachment of a drug. Polymethacrylates with trimethylammonium groups carrying salicylate anions (Sal^−^), which can be classified as the grafted and linear poly(ionic liquid)s (GPIL1 and LPIL1, respectively), demonstrated the release of pharmaceutical anions by ionic exchange with phosphate ones in buffer solution. Additionally, these polymers, as well as the analogical ones containing Cl^−^ (GPIL2, LPIL2), were self-assembled into the micellar carriers of non-ionic drugs, such as indomethacin (IMC) or erythromycin (ERY). Star-shaped polymethacrylates with *D*-glucopyranoside core were functionalized with carboxylic/amine groups (DGL1 and DGL2, respectively) to conjugate doxorubicin (DOX), which might be released by decomposition of hydrolysable covalent bonds. The polyacidic stars with pentaerythritol (PTL1-3) or *D*-glucopyranoside core (DGL3), including miktoarmed copolymers with extra poly(*ɛ*-caprolactone) (PCL) arms (DGL4), were also studied for encapsulation of the model drugs (DOX, IMC), which were delivered by polymeric micelles via diffusion process. The naming protocol of the discussed systems, for example GPIL1.1, consists of the symbol corresponding to the polymer group with the first number describing the series of polymers, and the second number identifying the sample in the series. In all these systems, the polymer composition and architecture can be used to adjust drug content and release properties, which were investigated to verify chemical potential of the prepared polymers as the drug carriers.

## 2. Non-Linear Polymers Containing Ionic Groups in Drug Delivery

In our studies, the atom transfer radical polymerization (ATRP) has been used to obtain the well-defined polymers with various topologies, i.e., grafted copolymers, as well as star copolymers, including V-shaped and miktoarmed structures (Figure 1). Pre-polymerization replacement of chloride anion in monomeric ionic liquid by pharmaceutical one, i.e., salicylate anion (Sal^−^), led to the design of the polymerized ionic drug carriers (DDS type I with ionically bonded drug). Post-polymerization modifications allowed for the introduction of specific functional groups (carboxyl or amine), which provided the possibility of chemical conjugation of the chosen drug (DDS type II with covalently bonded drug), whereas the induced amphiphilicity supported drug encapsulation (DDS type III with drug entrapped via physical interactions). The amphiphilic nature of macromolecules with diverse topology, including the grafted poly(ionic liquid)s with chloride anions, was beneficial in forming the self-assembling carriers in aqueous solution.

### 2.1. Poly(Ionic Liquid) Graft Copolymers (DDS Type I)

Previously designed by our group, amphiphilic ionic graft copolymers for DDS were based on anionic polyelectrolyte (polyacid) segments grafted from polymethacrylate backbone [30,31], or used as an extension of polyether (polyethylene or polypropylene glycol) side chains [32,33]. In aqueous solutions, depending on nature of side chains, they were self-assembled into different core–shell superstructures with ability for successful encapsulation of IMC [34,35,36].

The ionic properties are also represented by unique poly(ionic liquid)s, which are made of ionic monomers containing organic cation and organic or inorganic anion [37]. They gained great interest in material science because of macromolecular architectures, which can be tailored by combining both properties of cations and anions [38]. In our recent studies on the amphiphilic graft copolymers, the monomeric ionic liquids, i.e., commercially available (2-(methacryloyloxy)ethyl)-trimethylammonium chloride (ChMACl known as choline methacrylate) and containing pharmaceutical salicylate anion (ChMASal, [39,40]), were grafted from the standard multifunctional ATRP macroinitiators, i.e., poly(methyl methacrylate-*co*-(2-(2-bromoisobutyryloxy)ethyl methacrylate))) (poly(MMA-*co*-BIEM: MI) [41,42] with various contents of bromoester initiating groups, 25–75% (GPIL1 series and GPIL2, Figure 1c, Table 1). The side chains resulted in the use of methyl methacrylate (MMA) as the comonomer. It has been proved that polymers obtained from similar monomers, like phosphorylcholine methacrylate, provided low cytotoxicity [43]. The studies on polymers of ChMA indicated that depending on the grafting density, lengths of backbone, and side chains, the anionic drug content in the resulted cylindrical brushes can be tuned up to 40% weight of the polymer. In aqueous solution, the water-soluble macromolecules formed small superstructures with hydrodynamic diameters ranging from 20 to 60 nm. Low aggregation effect was probably caused by repulsive interactions between ionic moieties in the side chains, which yielded bigger particles with the increase in content of ionic units (and thus salicylate) at the same grafting degree (GPIL1.1 vs. GPIL1.2, GPIL1.3). However, the increase in grafting degree in polymers with the same content of ionic units resulted in particle size reduction (GPIL1.3 vs. GPIL1.4), although this difference was smaller when the ionic drug content was significantly higher due to larger grafting density (GPIL1.3 vs. GPIL1.5).

In the case of amphiphilic graft polymer with chloride anions (GPIL2.1), the therapeutic activity was introduced by self-assembling with encapsulation of nonionic drug, i.e., ERY or IMC. In a similar way, ERY was encapsulated by the salicylate-containing graft polymer (GPIL1.1) to form a dual drug system, but the content of encapsulated ERY was smaller than that for GPIL2.1 (6% vs. 20%). For comparison, the analogous linear poly(ionic liquid)s (LPIL1–2) were synthesized using standard ATRP initiator. i.e., ethyl α-bromoisobutyrate (EBiB) [39,40]. The LPIL systems, both chloride (LPIL2.1) and salicylate (LPIL1.1–1.2) ones in relation to analogical GPIL, exhibited higher drug loading content (DLC_LPIL_ > DLC_GPIL_), but this difference was significantly higher for salicylate systems, whereas the opposite DLC dependency was indicated for IMC encapsulation [44].

**Figure 2:**
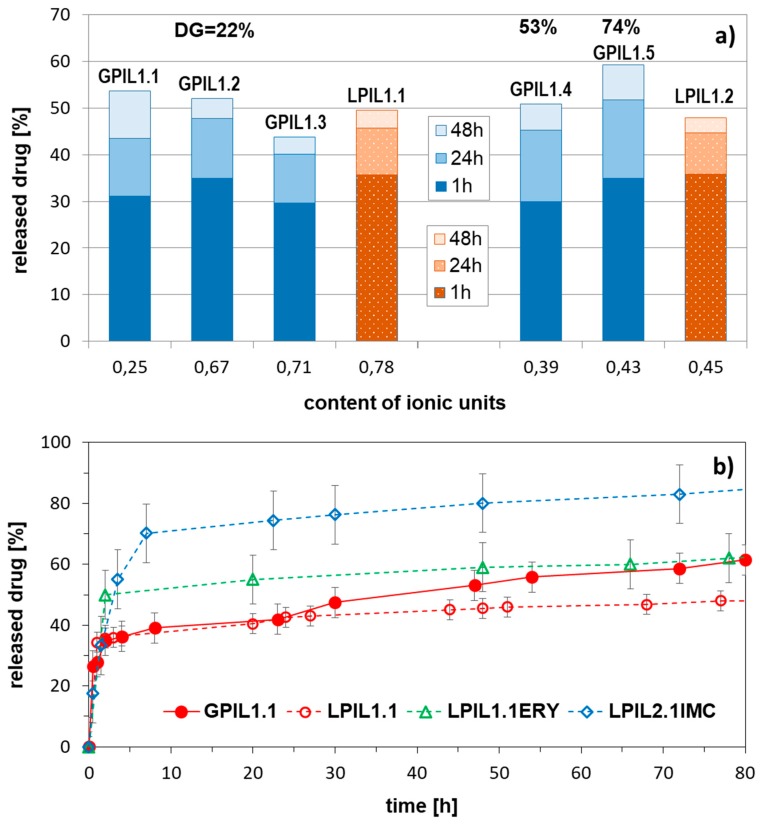
Amount of drug released after 48h in correlation with polymer topology (GPIL vs. LPIL), grafting degree (DG = 22–73%), and content of hydrophilic fraction (**a**), and release profiles of salicylate (GPIL1.1, LPIL1.1) and nonionic drugs (ERY, IMC) (**b**) in PBS at pH = 7.4 and 38 °C.

The ionic exchange was also postulated as the most probable mechanism of drug release. The dialysis experiments in PBS solution (pH = 7.4) indicated facile displacement of salicylate anions by phosphate ones, which represent better capability for coordination of the cations in polymer matrix. The burst release of the ionic drug attached to grafts can be explained by the dense packing character of grafting polymer topology, which intensified the repulsive interactions between negative charges on the aromatic rings of Sal^−^. Another advantageous ability of these ionic systems was good solubility of the polymer matrix in PBS environment after drug release. In the release studies there was no influence of the content of ionic units in side chains, when the copolymers with the same grafting degree were compared (Figure 2). However, at high grafting density corresponding to 3 grafts per 4 units in the backbone (GPIL1.5), the salicylate release was slightly accelerated.

Comparing the drug release profiles for the grafted poly(ionic liquid)s of ChMA (GPIL) and linear copolymer analogs (LPIL), there was no significant difference. Although, the content of salicylate anions ionically bonded to the polymer matrix was larger in the linear copolymers (Sal^−^ content 3.4–4.8 mg in 10 mg of linear polymer vs. 1.9–4.0 mg in 10 mg of graft polymer). According to the release studies approximately half of these drug amounts were exchanged and removed from the systems, that is 50% in the linear and 50–60% in the grafted carriers. The advantages for the latter ones were the size of particles, which were formed in aqueous solution. The largest particles of grafted polymers were 5–6 times smaller than the aggregates of linear analogs (56 nm vs. ~250 nm). These results suggested that the nonionic backbone was entangled into globular form with stretched stiff ionic side chains in the shell. Surprisingly, the release of a nonionic drug appeared to be troublesome, especially for the systems based on grafted copolymers (GPIL1.1 and GPIL2.1), where the release was not detected. The LPIL systems provided the miscellaneous release properties because LPIL1.1–1.2 containing Sal anions were able to release ERY, but this effect was not reached for chloride contained LPIL2.1, which supported IMC release (Figure 2). The lack of correlation can be explained by the system complexity related to carrier topology, possible repulsion effect of ionic groups, drug-polymer interactions, and anion bulkiness as responsible factors, which can cooperate providing extraordinary drug release behavior.

### 2.2. Conjugates of D-Glucopyranoside Based Star Copolymers (DDS Type II)

Star-shaped polymers with cleavable sugar core were obtained using di-, tri-, tetra-, hexakis(2-bromoisobutyrate) of mono-, and diacetal derivatives of *D*-glucopyranosides (DGL, *f* = 3–6) [45] as the multifunctional initiators in the controlled ATRP of methacrylates by core first technique. The resulting 2-, 3-, 4-, and 6-armed star copolymers containing protecting *tert*-butyl or reactive glycidyl groups, were modified by acidolysis (into methacrylic acid (MAA)) [46,47,48] or aminolysis (into 2-hydroxy-3-[(2-aminoethyl)amine]propyl methacrylate units (AmPMA)) [49] to introduce hydrophilic moieties, which in the next step were used as the sites for conjugation. Their contents were controlled by the length and number of arms, efficiency of the modification reaction, and by amount of the functionalized units having pendant carboxylic groups (polyacids, DGL1 series) [50] or amine groups (polybases, DGL2 series) [49,51] in the polyelectrolyte stars (Table 2, Figure 1a).

Previously, the conjugation for these stars has been performed with proper derivatives of fluorescein dye (amine in reaction with polyacids to form amide spacer or isothiocyanate with polybases through thiocarbamide bonding) [48,49], which was more successful for cationic than anionic polyelectrolytes (68% vs. 5%, respectively). Their cellular uptake studies by confocal laser scanning microscopy indicated that the fluorescent star polymers were found in the entire volume of cytoplasm, but the signal intensity received from polybases was stronger in comparison with polyacids, for which less effective internalization was caused probably by electrostatic repulsion with negatively charged cell membrane [49,52].

Presented polymer–drug conjugates were prepared from star-shaped polyacids (DGL1.1 – DGL1.4) and polybases (DGL2.1 – DGL2.4) via amide or ketoimine linking DOX, respectively [52,53]. Various polymeric prodrugs with DOX have been investigated by other groups [54,55,56,57,58], to reduce its well-known severe side effects. Our approach was to take advantage of several aspects in the structure of the polymeric carrier, such as sugar-derived biodegradable core, cleavable amphiphilic arms, decreased hydrodynamic volume in solution in comparison with linear analogues, increased effectiveness of the drug protection, and longer time of circulation in the blood stream. Comparing drug conjugation with 4-armed stars based on polybases and polyacids with equimolar content of hydrophilic fraction higher efficiency was observed for formation of ketimine than amide bond (64% at n_DOX_ = 59 in DGL2.3 vs. 4% at n_DOX_ = 2 in DGL1.1) similarly to the fluorescein conjugates. Moreover, the conjugation efficiency of polybases decreased with the increase of amine repeating units per arm, whereas in the case of polyacids, the amount of attached drug increased with the number of the arms as well as the content of acidic units.

**Figure 3:**
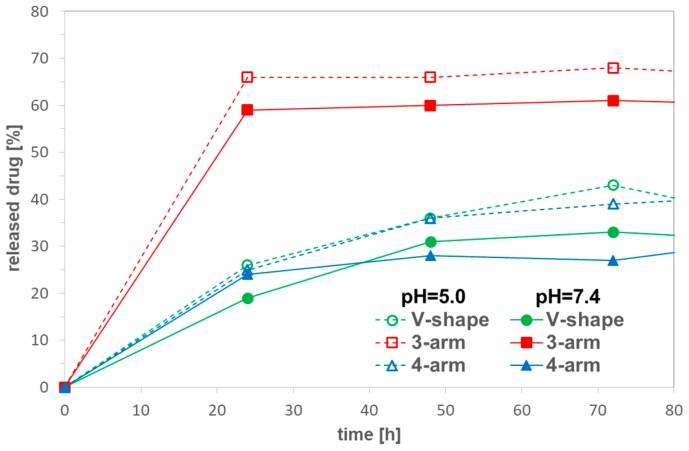
DOX release profiles from conjugates of star-shaped copolymers with equimolar compositions and various number of arms, DGL2.1 (V-shaped), DGL2.2 (3-armed), and DGL2.3 (4-armed) in PBS at 37 °C.

The release studies were performed only for polymeric prodrugs based on polybases, due to the poor solubility of polyacid-DOX conjugates in water. The lowest amount of drug was released by conjugate, based on 4-armed star (DGL2.3), whereas 3-armed system (DGL2.2) was able to supply release of twice larger drug doses (Figure 3). In acidic conditions (pH 5.0), which are more favorable for hydrolysis of ketimine group than the neutral pH (0.01M PBS, pH 7.4), the drug release occurred faster.

### 2.3. Self-Assembling Star-Shaped Copolymers (DDS Type III)

Another group of 4-armed stars was synthesized in a similar way to sugar based stars using a core-first strategy via ATRP initiated by tetrakis(2-bromoisobutyrate) of pentaerythritol (PTL, *f* = 4), and then acidolysis of *tert*-butyl groups in the copolymers to deprotect carboxylic groups (PTL1-3 series, Figure 1b, Table 3) [47]. Combinations of two methacrylates (MAA and MMA, PTL1) or methacrylate and acrylate (MAA and methyl acrylate (MA) as PTL2, MMA and acrylic acid (AA) as PTL3) with various proportions in the arms were investigated to form the self-assembling PTL cored star copolymers as the micellar carriers of IMC.

**Figure 4:**
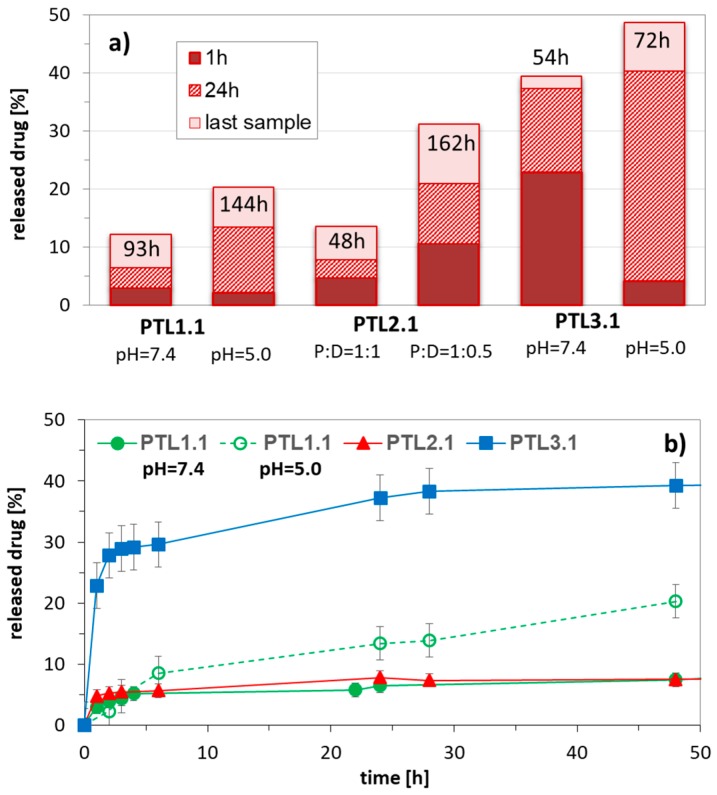
Influence of pH and weight ratio of polymer to encapsulated drug on amount of released drug in correlation with composition of 4-armed copolymers of MMA/MAA (PTL1.1), MMA/AA (PTL2.1), and MA/MAA (PTL3.1) with equimolar content of hydrophilic fraction (**a**), and representative release profiles of IMC (**b**) in PBS at 37 °C.

The adjustable distribution of acidic units was convenient for controlling the contents of hydrophilic fraction, which affected the efficiency of drug encapsulation and release. The highest drug loading content was obtained for copolymers with equimolar compositions (DLC = 50–90% (50/50)). The copolymers of MMA/MAA and MMA/AA, with comparable amounts of hydrophilic fractions (PTL1.1 and PTL2.1), exhibited formation of aggregates at the same concentrations, whereas CMC for MA/MAA system (PTL3.1) was twice as high in comparison with MMA copolymers (0.030 vs. 0.017 mg/mL). The rate of drug release for these systems can be summarized by the following order: MMA/MAA < MMA/AA << MA/MAA, which shows strong influence of arm composition. During release studies we have noticed that within 1 h the drug was released faster in neutral conditions than in an acidic environment, whereas after longer time this tendency was reversed (Figure 4). Additionally, it was detected that the reduced drug release can also be forced by increased encapsulation ratio of drug to polymer as it is presented for PTL2.2 (MMA/AA system).

Amphiphilic character of star copolymers bearing hexakis(2-bromoisobutyrate)-dihydroxy-*D*-(–)-salicin core (DGL3 series, Figure 1b, Table 3) [46,48] gave the opportunity to form more or less globular self-assembling systems in aqueous solution depending on ionization degree of statistically distributed ionizable hydrophilic units in the arms. However, the hydrophilic–hydrophobic balance can also be shifted by introduction of additional hydrophobic arms. In our studies, the miktostars (DGL4 series, Figure 1b, Table 3) [46] were obtained using two unprotected hydroxyl groups in the core of polymethacrylate-based macroinitiator (DGL3) as the initiating sites in the insertion-coordination ring opening polymerization (ROP) catalyzed by tin(II) bis(2-ethylhexanoate). Combined with amphiphilic polymethacrylate arms containing acidic groups, the formed two poly(ε-caprolactone) (PCL) arms provided phase-separation in aqueous solution. The yielded micelles with biodegradable core were detected as bigger particles than their amphiphilic polymethacrylate precursors, playing the role of the bifunctional macroinitiators in ROP, for example DGL3.2 vs. DGL4.2.

The self-assembly studies on polyacidic stars DGL3 with a similar length of polymethacrylic arms (DP_arm_ ~ 50) has revealed that CMC drastically decreased with the increase in hydrophilic content in the arms (0.172, 0.024, and 0.006 mg/mL, respectively). Comparing 6-armed polymethacrylate stars and their miktoarm analogues (with extra two hydrophobic PCL arms), the aggregation of the latter ones was not dependent on the hydrophilic–hydrophobic ratio (CMC of DGL4.1 = DGL4.2 = 0.030 mg/mL).

**Figure 5:**
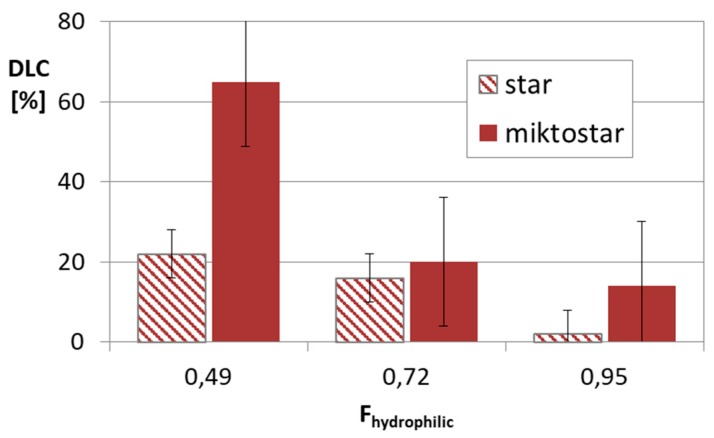
Drug content in micellar systems based on amphiphilic star (DGL3 series) and miktostar (DGL4 series) copolymers, where F_hydrophilic_ is average value for comparable pair of star and miktostar.

**Figure 6:**
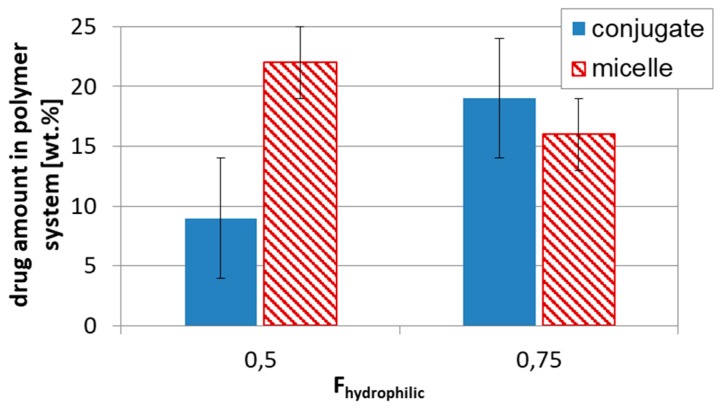
Influence of drug loading type, conjugates DGL1 vs. micelles DGL3, on drug content in respect to hydrophilic–hydrophobic balance.

The successful aggregation of sugar-cored polyacidic stars encouraged us to provide systems with encapsulated DOX (≤ 65% at polymer/drug ratio = 1:0.5). The drug loading content was reduced with the increase in arm length and the same with the hydrophilic content in the micellar carriers, but comparing stars with the corresponding miktostars, the latter ones seemed to be more promising carriers, especially those with the equimolar compositions (Figure 5). It is also worth noticing that the 6-armed acidic copolymers with similar arm lengths and equimolar compositions were able to entrap physically larger amounts of DOX than it was chemically conjugated (22% in DGL3.1 vs. 9 wt% in DGL1.3 at DP_arm_ = 60), but in more hydrophilic systems this difference was insignificant (16% in DGL3.2 vs. 19 wt% in DGL1.4 at DP_arm_ = 50) (Figure 6).

**Figure 7:**
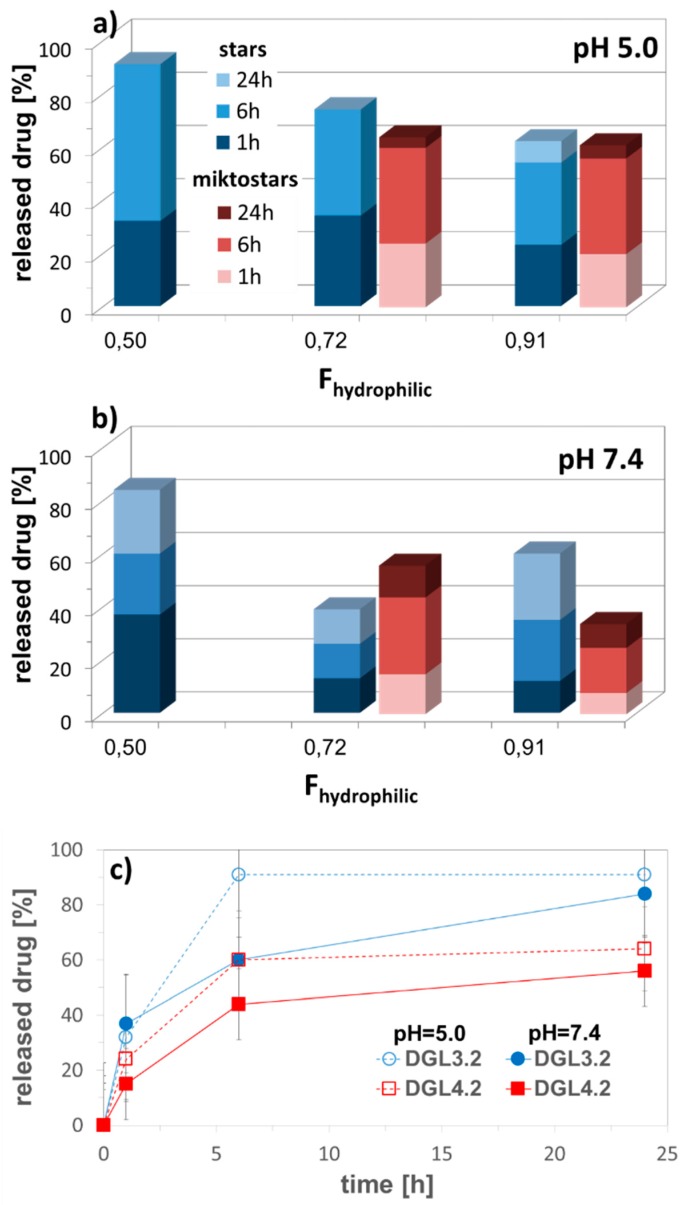
Amount of released DOX from self-assembled star DGL3 vs. miktostar DGL4 copolymers in correlation with content of hydrophilic fraction in acidic and neutral conditions (**a**,**b**), and representative release profiles of encapsulated DOX (**c**) in PBS at 37 °C.

The equimolar system DGL3.1 has also provided the fastest drug release. No significant difference in percentage amount of released drug between star and miktostar analogs was observed at pH 5, but in neutral solution the miktostar with dominating hydrophilic fraction (DGL4.2) was more efficient than the analogous star (DGL3.2), which was in contrast to the more hydrophilic systems DGL4.3 vs. DGL3.3 (Figure 7). Another comparison of drug release from polybase-DOX conjugates (DGL2) and polyacid based micelles loaded with DOX (DGL3, DGL4), indicated in almost all cases that the drug was released faster within first 6 h (for micelles) and 24 h (for polymer-DOX conjugates) at acidic environment than at neutral pH. For example, 60% of DOX was delivered at pH = 5.0 and 44% at pH = 7.4 by DGL4.3, whereas 84% and 56% by DGL2.1, respectively. It is highly probable that the destabilization of self-assembly containing carboxylic groups in the outer layer was activated by the ionized DOX with amine groups, which are protonated in acidic conditions.

## 3. Drug Distribution and Cytotoxicity

The mathematical models describing the kinetics of drug release suggested the diffusion mechanism. It was confirmed by the good agreement with the Higuchi model represented as the plot of the cumulative amount of released drug against the square root of time (correlation coefficient R^2^ = 0.90–0.99) for the release of ionic and nonionic drugs (ERY, IMC) from LPIL and GPIL systems [42,44]. Additionally, the kinetics of the nonionic drug release from LPIL1, LPIL2, and GPIL2 systems was also concentration dependent, showing good fit with the first-order kinetic model (R^2^ = 0.93–0.98) [44], which is expressed by a logarithm of the percentage of drug remaining vs. time. Similarly, the concentration dependent, and diffusion controlled, release of IMC was reported for the self-assembling graft copolymers with PMAA side chains (the first-order kinetic model R^2^ = 0.9–0.99 and Higuchi model R^2^ = 0.9–0.97) [34,36], as well as the linear block copolymers PCL-*b*-PMAA, and their three armed stars (the first-order kinetic model R^2^ = 0.85–0.99 and Higuchi model R^2^ = 0.91–0.99) [59] or stars with PTL core (the first-order kinetic model R^2^ = 0.87–0.99 and Higuchi model R^2^ = 0.9–0.99) [47]. In the case of conjugate systems DGL2-DOX the Korsmeyer–Peppas model, based on the diffusion exponent n, which describes Fickian (*n* ≤ 0.45) and non-Fickian (0.45 < *n* < 0.89), the release of drug was applied for verification of release mechanism. According to the “n” values, almost all samples followed the Fickian diffusion (*n* < 0.45, R^2^ = 0.928−0.999) [51].

**Figure 8:**
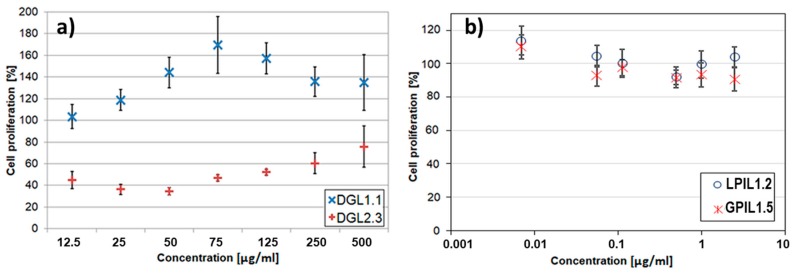
In vitro cytotoxicity effect of polymethacrylate carriers containing carboxylic (DGL1.1) or amine groups (DGL2.3) without drug on MCF-7/R cells (**a**), and trimethylammonium groups with salicylate anions (GPIL1.5, LPIL1.2) towards BEAS-2B cells (**b**).

In respect to DDS applications, the cytotoxicity of designed copolymers was verified by viability of the selected cell lines. The human bronchial epithelial cells (BEAS-2B) were applied for both linear and graft copolymers with trimethylammonium groups and salicylate counterions, which due to their anti-inflammatory and antibacterial activity potential can be beneficial in the treatment of lung and bronchi diseases [42]. In vitro studies evaluated by MTT assay exhibited very low cytotoxicity towards BEAS-2B as it is shown in Figure 8. Generally, the cells treated with GPIL5 showed slightly lower viability than LPIL1.2. However, at concentration 0.0025 µg/mL both LPIL1.2 and GPIL1.5 stimulated cell growth, which was depicted by cell proliferation at levels of 114% and 110% in comparison to the control, respectively. In the case of polymers functionalized with carboxylic or amine groups, the influence of charged polymer particles on their interactions with cells was evaluated by MTS tests using DOX-resistant breast cancer cells (MCF-7/R), because the star-shaped copolymers with DGL core were prepared for conjugation or encapsulation of anticancer cytostatic DOX. The results for MCF-7/R treated with representative drug-free, 4-armed, star-shaped polyacid DGL1.1 showed significant cytotoxicity in comparison to the polybase DGL2.3 with the same number of arms and hydrophilic content (Figure 8). However, cytotoxicity of polybasic carriers increased with a decrease in the number of arms DGL2.1 > DGL2.2 > DGL2.3 [53], whereas 6-armed polyacids were statistically less cytotoxic than their 4-armed analogs [52]. Moreover, polyacids did not display changes in viability of colon cancer cells (HCT-116) and viability of normal human dermal fibroblasts (NHDF) cells, which stayed at the acceptable level [52]. These results are in contrast to those obtained for polybases, which showed inhibition of HCT-116 cells proliferation and low cytotoxicity toward NHDF cells [49].

## 4. Experimental

### 4.1. Characterization Techniques

Molecular weights and dispersity indices (Ð) were determined by size exclusion chromatograph (SEC, 1100 Agilent 1260 Infinity) equipped with an isocratic pump, autosampler, degasser, thermostatic box for columns (PLGel 5 mm MIXED-C 300 7.5 mm and pre-column guard 5 mm × 7.5 mm), and differential refractometer MDS RI Detector. Addon Rev. B.01.02 data analysis software (Agilent Technologies) was used for data collecting and processing. The calculation of molecular weight was based on calibration using linear polystyrene standards (580–300,000 g/mol). The measurements were carried out in THF or DMF (HPLC grade) as the solvent at 40 °C with flow rate of 0.8 mL/min.

^1^H NMR spectra of copolymers in CDCl_3_, DMSO-d_6,_ or D_2_O were collected on Varian Inova 600 MHz spectrometer at 25 °C using appropriate internal standard (TMS or TSP).

The hydrodynamic diameters (D_h_) of particles were measured by dynamic light scattering (DLS) using a Malvern Zetasizer. Samples placed in PMMA cell after appropriate dilution with a solvent (0.2, 0.4, 0.5, or 1 mg/mL) were put in the thermostatted cell compartment of the instrument at 25 °C.

The CMC was measured by fluorescence spectrophotometry (Hitachi F-2500) using pyrene as fluorescence probe. Excitation spectra of pyrene (λ = 390 nm) were recorded at constant concentration of pyrene (3.0 × 10^−4^ mol/L) and polymer concentrations in the range of 5 × 10^−4^–1.0 mg/mL. The intensity ratio (I_336_/I_332_) from pyrene excitation spectrum vs. logC (where C is concentration in mg/mL) was plotted, where the cross-over point was estimated as the CMC value.

For the determination of drug content, polymer systems were dissolved in H_2_O under vigorous vortexing and analyzed using a UV–Vis spectrophotometer (Thermo Scientific Evolution 300) at 480 nm (DOX), 320 nm (IMC). Calibration curves were obtained for drug-H_2_O solutions with different drugs concentrations. Drug loading content (DLC for micelles), or drug content (DC for conjugates) and drug loading efficiency (DLE) were calculated using the following equations:

(1) $$DLC=\frac{\mathrm{weight}\;\mathrm{of}\;\mathrm{drug}\;\mathrm{loaded}\;\mathrm{into}\;\mathrm{micelle}\;}{\mathrm{total}\;\mathrm{weight}\;\mathrm{of}\;\mathrm{polymer}\;\mathrm{and}\;\mathrm{loaded}\;\mathrm{drug}\;}\times 100\%$$

(2) $$DC=\frac{\mathrm{weight}\;\mathrm{of}\;\mathrm{drug}\;\mathrm{in}\;\mathrm{conjugate}\;}{\mathrm{weight}\;\mathrm{of}\;\mathrm{drug}-\mathrm{polymer}\;\mathrm{conjugate}}\times 100\%$$

(3) $$DLE=\frac{\mathrm{weight}\;\mathrm{of}\;\mathrm{drug}\;\mathrm{loaded}\;\mathrm{into}\;\mathrm{micelle}\;}{\mathrm{weight}\;\mathrm{of}\;\mathrm{drug}\;\mathrm{in}\;\mathrm{feed}\;}\times 100\%.$$

In vitro drug release studies were performed in 0.01 M PBS at pH 7.4 or 5.0 (the pH of 0.01 M PBS was subsequently adjusted with 0.1 N HCl to pH 5.0). The lyophilized drug-contained system (2.0 mg) was dissolved in PBS (2.0 mL) and transferred into a dialysis bag (MWCO of 3.5 kDa, Spectrum Laboratories Inc). Then, the dialysis bag was immersed into PBS (20.0 mL) at 37 °C and stirred. At predetermined time intervals, 1 mL of the buffer solution outside the dialysis bag was taken out. UV–vis spectroscopy (Thermo Scientific Evolution 300) was used to determine the amount of released drug by measuring the absorbance maximum (NaSal at 295–298 nm, ERY at 285 nm, IMC at 320 nm, and DOX at 480 nm). Each result is an average of three parallel measurements.

### 4.2. Cell Viability Assessment

In vitro cytotoxicity of the selected copolymers was measured using the MTS or MTT assay (Promega cell proliferation assays) and following cell lines: Bronchial epithelial cells (BEAS-2B from ATCC, Manassas, VA, USA), human colon cancer cells, and human breast cancer cells resistant to DOX (HCT116 and MCF-7/R from ATCC as a kind gift from the Center of Oncology – Maria Sklodowska-Curie Memorial Institute).

Briefly, the selected cells were seeded in a 96-well micro titer plates at a density of 10,000 cells per well and incubated overnight at 37 °C. All cells were grown in DMEMF12 medium (Sigma-Aldrich, Germany), supplemented with 10% (v/v) inactivated fetal bovine serum (FBS) (EURx, Poland) and 1% antibiotics (10,000 μg/mL of streptomycin and 10,000 units/mL of penicillin) (Sigma-Aldrich, Germany), at 37 °C in humidified atmosphere with 5% CO_2_. After 24 h of incubation under standard conditions, a series of suspension dilutions were added into wells. The cytotoxicity was evaluated after a predetermined time of incubation (24 or 72 h). The absorbance at 490 nm was measured using a microplate reader (Epoch, Biotek, Winooski, VT, USA). All experiments were performed in quadruplicate, and the relative cell proliferation (%) was expressed as a percentage relative to the untreated control cells (positive control). For more details, see ref. [24,36,43].

## 5. Conclusions

The nonlinear amphiphilic polymers, including star and graft topologies, are attractive nanocarriers for drug delivery because, similarly to linear macromolecules, they are capable of transporting drugs. The bioactive compounds can be effectively introduced into polymer matrix via chemical bonding to form drug-polymer conjugates, including poly(ionic liquid)s with pharmaceutical counterions, or by physical interactions in the self-assemblies. Due to the extra structural parameters related to topology, e.g., number of arms/grafts, the nonlinear polymers offer much broader range of drug loading and release moderation. The reviewed polymeric systems varying with types of drug loading (physical vs. covalent vs. ionic) were represented by nanoparticles with sizes up to 200 nm, with exception of miktostars (up to 500 nm). Although the grafted copolymers were able to carry similar amounts of ionically attached drug as their linear analogs, they are still advantageous systems for delivery due to smaller sizes of nanoparticles. Moreover, the design of DDS combining different strengths of drug entrapment can be strategic for sequential drug release in the combined therapy. Our studies confirmed that knowing the general correlations between structure, and loading/release effect for the obtained polymers, the nanocarrier activity with controllable pharmacokinetic properties can be properly regulated.

## Figures and Tables

**Table 1:** Characterization of poly(ionic liquid)s GPIL vs. LPIL (DDS type I).

No.	n_sc_^a^	F_ChMAX_^b^ (mol %)	DP_sc_^c^	DG ^d^ (%)	M_n,NMR_ (g/mol)	M_n,SEC_^e^ (g/mol)	Ð ^e^	D_h_^f^ (nm)	DC (%)	Ref.
GPIL1.1	75	25	24	22	201,500	17,680	1.04	28	19	[44]
									6^g^	
GPIL1.2	75	67	12	22	127,000	19,500	1.04	56	32	
GPIL1.3	75	71	28	22	314,500	19,000	1.10	51	36	
GPIL1.4	105	74	39	53	724,100	30,400	1.06	22	38	
GPIL1.5	185	74	43	74	1,498,500	828,700	1.28	40	39	
LPIL1.1	-	78	187 *	-	49,200	6200	1.25	293	41	[39,40]
									49 ^g^	[42]
LPIL1.2	-	45	119 *	-	23,100	8300	1.42	232	32	[39,40]
									51 ^g^	[42]
GPIL2.1	165	19	26	53	579,000	nd	nd	24	20 ^g^	[44]
									32 ^h^	
LPIL2.1	-	26	233 *	-	28,000	10,400	1.36	149	45 ^g^	[42]
									11 ^h^	[42]

GPIL: grafted poly(ionic liquid)s, where GPIL1: poly(MMA-*co*-(BIEM-*graft*-P(MMA-*co*-ChMASal)), GPIL2: poly(MMA-*co*-(BIEM-*graft*-P(MMA-*co*-ChMACl)); LPIL: linear poly(ionic liquid), where LPIL1: P(MMA-*co*-ChMASal), LPIL2: P(MMA-*co*-ChMACl); ^a^ number of side chains, ^b^ content of ionic units in polymer, ^c^ degree of polymerization of side chains, ^d^ degree of grafting related to n_sc_ per total DP of backbone, ^e^ determined in DMF, ^f^ determined in deionized water, ^g^ DLC of ERY for the weight ratio of polymer to encapsulated drug P:D = 1:1; ^h^ DLC of IMC for the weight ratio of P:D = 1:1; * DP of LPIL; nd – not determined.

**Table 2:** Characterization of star-shaped polyacids DGL1 and polybases DGL2 (DDS type II).

No.	Polyelectrolytes							DOX Conjugates		ref.
	*f*	F_h-philic_^a^	DP_arm_^b^	M_n,NMR_ (g/mol)	M_n,SEC_^c^ (g/mol)	Đ ^c^	D_h_^d^ (nm)	DC (%)	D_h_^d^ (nm)	
DGL1.1	4	0.56	58	21,800	11,800	1.17	10	5	insoluble	[48,52]
DGL1.2	4	0.74	68	24,200	10,900	1.28	8	14	insoluble	
DGL1.3	6	0.51	62	35,600	16,300	1.20	9	6	insoluble	
DGL1.4	6	0.75	54	30,100	insoluble	insoluble	8	19	insoluble	
DGL2.1	2	0.54	51	15,800	nd	nd	8	27	8	[49,51]
DGL2.2	3	0.49	57	26,500	nd	nd	7	28	8	
DGL2.3	4	0.53	65	41,100	nd	nd	12	17	11	
DGL2.4	4	0.77	52	38,000	nd	nd	12	24	12	

DGL star-shaped polymer with D-glucopyranoside core, where DGL1: *s*-P(MMA-*co*-MAA)_f_, DGL2: *s*-P(MMA-*co*-AmPMA)_f_; ^a^ content of hydrophilic fraction in the polymer, ^b^ degree of polymerization of arm, ^c^ determined in THF, ^d^ determined in PBS solution 0.4 mg/mL; *f:* number of arms; nd: not determined.

**Table 3:** Characterization of star-shaped polyacids used for drug encapsulation (DDS type III).

Polyacids	Polyelectrolytes							Drug-Loaded		ref.
	*f*	F_h-philic_^a^	DP_arm_^b^	M_n,NMR_ (g/mol)	M_n,SEC_^c^ (g/mol)	D ^c^	D_h_^d^ (nm)	DLE ^h^ (%)	D_h_^d^ (nm)	
PTL1.1	4	0.48	34	15,400	11,600	1.17	147 ^e^	74	571	[47]
PTL2.1		0.36	63	27,800	18,200	1.32	198 ^f^	48	628	
PTL2.2		0.70	67	31,800	16,800	1.31	161 ^f^	6	463	
PTL3.1		0.55	56	27,000	17,700	1.30	198 ^f^	86	874	
PTL3.2		0.76	39	20,200	13,600	1.28	162 ^f^	9	579	
PTL3.3		0.98	31	17,400	11,500	1.24	114 ^g^	7	731	
DGL3.1	6	0.50	55	32,100	insoluble	insoluble	202	66	531	[46,48]
DGL3.2		0.75	50	28,700	6000	1.33	165	47	321	
DGL3.3		0.97	47	25,700	insoluble	insoluble	180	7	1165	
DGL4.1	8 (6 + 2) *	0.48	54/19 *	28,700	8400	1.57	517	48	705	[46]
DGL4.2		0.69	44/10 *	28,900	4000	1.67	252	42	1169	
DGL4.3		0.92	65/10 *	41,900	6600	1.44	384	60	> 10,000	

PTL: Star-shaped polymer with pentaerythritol core, where PTL1: *s*-P(MMA-*co*-MAA), PTL2: *s*-P(MMA-*co*-AA), PTL3: *s*-P(MA-*co*-MAA); DGL: Star-shaped polymer with D-glucopyranoside core, where DGL3: *s*-P(MMA-*co*-MAA)_6_, DGL4: *s*-P(MMA-*co*-MAA)_6_PCL_2_; ^a^ content of hydrophilic fraction in the polymer, ^b^ degree polymerization of arm, ^c^ determined in THF, ^d^ determined in PBS solutions 0.4 mg/mL, ^e^ 0.5 mg/mL, ^f^ 0.2 mg/mL, ^g^ 1 mg/mL; ^h^ for the weight ratio of polymer to encapsulated drug P:D = 1:1 (PTL) and 2:1 (DGL); * P(MMA-*co*-MAA) and PCL arms, respectively.
